# Fungal CO_2_ Fixation in Soil Systems: Distinct Roles of PEPC and PC in Carbon Allocation Under Varying CO_2_ and Thiosulfate Conditions

**DOI:** 10.3390/jof12100717

**Published:** 2026-09-24

**Authors:** Jiji Wang, Shuwen Guo, Qiaogai Lu, Ankang Wang, Anning Yang, Yue Wang, Zhanhui Zhao, Shiying Li, Mingyu Wu, Fang Li

**Affiliations:** 1 State Key Laboratory of High-Efficiency Production for Wheat-Maize Double Cropping System, College of Resources and Environment Science, Henan Agricultural University, Zhengzhou 450046, China; 2 School of Surveying and Urban Spatial Information, Henan University of Urban Construction, Pingdingshan 467036, China

**Keywords:** fungal CO_2_ fixation, elevated CO_2_, ^13^C labeling, anaplerotic carbon fixation

## Abstract

The role of filamentous fungi in soil CO_2_ fixation under elevated CO_2_ concentrations remains poorly understood, particularly regarding the regulatory mechanisms of CO_2_ fixation enzymes. In this study, a microcosm incubation experiment was conducted to investigate the effects of Glucose, Na_2_S_2_O_3_, and CO_2_ concentrations on the CO_2_ fixation capacity of *Trichocladium uniseriatum*. A full-factorial design with three Glucose levels (0, 100, and 200 mg·kg^−1^), three Na_2_S_2_O_3_ levels (0, 50, and 100 mg·kg^−1^), and two ^13^CO_2_ levels (5% and 10%) was employed. Fungal colony growth, activities of phosphoenolpyruvate carboxylase (PEPC) and pyruvate carboxylase (PC), and the accumulation of ^13^C in different soil carbon fractions were analyzed. Results showed that colony diameter was significantly influenced by Glucose and Na_2_S_2_O_3_ concentrations, with optimal growth observed under moderate levels (100 mg·kg^−1^ Glucose and 50 mg·kg^−1^ Na_2_S_2_O_3_) at 10% CO_2_. PC activity was negatively correlated with CO_2_ concentration but positively correlated with Na_2_S_2_O_3_ concentration, whereas PEPC activity showed positive correlations with both CO_2_ and Na_2_S_2_O_3_. The δ^13^C abundance in active carbon fractions (dissolved organic carbon, DOC, and microbial biomass carbon, MBC) was significantly higher than in soil organic carbon(SOC), with MBC showing the greatest enrichment. Random forest models identified distinct drivers for ^13^C accumulation across carbon fractions: CO_2_ and PEPC contributed to ^13^C accumulation in SOC, while PC activity drove ^13^C allocation to DOC and MBC. Partial least squares path modeling further revealed that PC and PEPC collectively mediated ^13^C allocation to DOC, with PC specifically driving ^13^C into MBC and PEPC channeling ^13^C into SOC. These findings demonstrate that *T. uniseriatum* mediates CO_2_ fixation through the differential regulation of PEPC and PC, thereby influencing the partitioning of fixed ^13^C among soil carbon fractions. This study provides mechanistic insights into fungal-driven CO_2_ fixation under changing atmospheric CO_2_ scenarios.

## 1. Introduction

Rising atmospheric CO_2_ levels exacerbate climate risks. As the largest carbon reservoir on land, soil absorbs approximately 10% of atmospheric carbon annually, making it a critical component for achieving carbon peaking and carbon neutrality goals [1]. Currently, terrestrial CO_2_ release contributes to about 60% of the increase in atmospheric CO_2_ concentration [2], highlighting the importance of enhancing soil carbon sequestration. Soil microorganisms are central drivers of the soil carbon cycle. The conceptual framework of the “soil microbial carbon pump” (SMCP) describes how heterotrophic microbes regulate soil carbon dynamics through “in vivo modification” and “ex vivo turnover”, while relying on the “entombing effect” to stabilize microbial residual carbon [3]. In recent years, the updated “SMCP conceptual framework 2.0” has incorporated autotrophic microbial carbon fixation pathways, illustrating how microorganisms can directly assimilate atmospheric CO_2_ through six distinct fixation routes, significantly boosting soil carbon sink capacity [4,5]. Studies show that the abundance of autotrophic microorganisms is strongly correlated with CO_2_ uptake rates, underscoring their key role in enhancing soil carbon sequestration potential [6]. These microbes are widely distributed across diverse soil ecosystems, including croplands, wetlands, grasslands, forests, paddy fields, and even deserts. Their carbon fixation rates range from 5.57 to 85.1 mg C/m^2^/day, continuously replenishing the soil carbon pool [7]. Estimates indicate that in forest, dryland, and paddy soils, microbial CO_2_ fixation accounts for approximately 0.45%, 0.48%, and 1.12% of soil organic carbon annually, with topsoil (0–20 cm) sequestration reaching 142–194, 128–174, and 536–731 kg·ha^−1^ per year, respectively [8]. Even in unfertilized chernozem soils, autotrophic microbes can assimilate CO_2_ at a rate of 0.4 mg·kg^−1^/day [9]. Furthermore, autotrophic microorganisms play a crucial role in SOC accumulation in degraded soils. For instance, in soil erosion areas of the Loess Plateau, microbial CO_2_ assimilation reaches 0.839–5.568 mg C/km^2^/year [10], while CO_2_ fixation rates in mildly and severely degraded soils are 2.15 and 1.52 times higher, respectively, than in non-degraded soils [11]. Collectively, this evidence underscores the significant potential of microbial carbon fixation in leveraging CO_2_ to enhance soil carbon stocks, particularly in low-fertility soils.

Current research on soil CO_2_ fixation primarily focuses on autotrophic bacteria and archaea, with studies on fungal CO_2_ fixation being extremely limited [12]. However, elevated atmospheric CO_2_ concentrations can shift energy flow more toward fungal pathways than bacterial pathways [13]. In the topsoil (0–30 cm), the global fungal biomass carbon budget is estimated at 12 Pg C, three times that of bacteria [14]. Fungi contribute more significantly to CO_2_ fixation in nutrient-poor soils, whereas bacteria dominate in carbon-rich soils [15]. Fungi can fix carbon through both heterotrophic and autotrophic pathways: heterotrophic fixation mainly relies on pyruvate carboxylase (PC) and phosphoenolpyruvate carboxylase (PEPC) in the anaplerotic reactions of the tricarboxylic acid cycle, whose activities are significantly affected by elevated CO_2_ concentrations [16]. Simultaneously, organic carbon sources (e.g., Glucose) can influence this process by modulating central carbon metabolism [17]. Autotrophic CO_2_ fixation in fungi is exceptionally rare. Early studies based on ^14^C labeling experiments suggested that fungi can fully rely on CO_2_ for carbon assimilation and found that reduced sulfur compounds (e.g., Na_2_S_2_O_3_) effectively stimulate their autotrophic growth [18,19]. Our previous research, using phospholipid fatty acid stable isotope probing across 29 sites in China uplands, revealed that fungal CO_2_ fixation accounts for 2.2–65.5% of total microbial CO_2_ fixation. Moreover, the synthesis of melanin enhances the recalcitrance of their residues, prolonging their retention in soil [15,20].

The saprophytic fungus *Trichocladium uniseriatum* (*T. uniseriatum*) has been widely documented for its high efficiency in degrading recalcitrant carbon compounds such as cellulose and lignin [16,17]. Notably, beyond its role in decomposition, its significant autotrophic CO_2_ assimilation capacity has been confirmed, with an estimated annual carbon fixation potential of approximately 0.008–0.01 Pg C [15]. Crucially, over 80% of the ^13^C fixed by *T. uniseriatum* enters the long-lived mineral-associated organic carbon pool via the microbial biomass carbon (MBC) pool, underscoring its ecological importance in soil carbon sequestration [15]. Genomic analysis has identified up to 46 CO_2_ fixation-related genes in *T. uniseriatum*, with the expression of the autotrophic ATP citrate lyase gene and the sulfite oxidase gene (which supplies energy) being regulated by SO_3_^2−^ addition. However, the contributions of the anaplerotic key enzymes PC and PEPC to CO_2_ fixation, as well as their regulation by carbon sources, reducing sulfur, and CO_2_ concentration, remain unclear. We hypothesize that the CO_2_ fixation rate of *T. uniseriatum* and the partitioning of fixed carbon among soil carbon pools are jointly regulated by the interactive effects of atmospheric CO_2_ concentration, exogenous organic carbon (e.g., Glucose), and reducing substances (e.g., Na_2_S_2_O_3_). To test this hypothesis, we conducted a soil microcosm incubation experiment by inoculating *T. uniseriatum* under a full-factorial design varying CO_2_ level, Glucose addition, and Na_2_S_2_O_3_ supplementation. This study aims to systematically elucidate how these three key environmental factors regulate the fungal growth dynamics, CO_2_ fixation, and ultimately, the allocation of fixed carbon into different soil carbon pools. Our findings will provide mechanistic insights into fungal-driven soil carbon sequestration processes.

## 2. Materials and Methods

### 2.1. Experimental Materials

The tested soil was a typical cultivated loamy fluvo-aquic soil (26% clay; Aquic Inceptisol, USDA Soil Taxonomy) derived from the alluvial sediments of the Yellow River. It was collected from the experimental station of the Henan Academy of Agricultural Sciences (Yuanyang, 34°47′ N, 113°40′ E, Henan Province, China). After removing the surface litter layer, the soil was sampled at a depth of 0–20 cm. The basic physicochemical properties of the soil were as follows: pH 8.2, organic carbon 14.6 g·kg^−1^, total nitrogen 0.65 g·kg^−1^, available phosphorus 15 mg·kg^−1^, and available potassium 128 mg·kg^−1^. The collected soil was sieved through a 2 mm mesh to remove plant and animal residues, stones, and other impurities. After thorough mixing and air-drying, it was stored for later use. The *T. uniseriatum* strain was isolated and preserved by our laboratory, with the preservation number CGMCC:18156.

Potato dextrose agar (PDA) medium: 200 g of peeled potatoes were sliced, boiled in distilled water, and the extract was filtered through eight layers of gauze. Then, 20 g of Glucose and 15 g of agar were added to the extract. The volume was adjusted to 1000 mL with distilled water. After dispensing into Erlenmeyer flasks, the medium was sterilized by autoclaving at 121 °C for 20 min. It was poured into 9 cm Petri dishes under sterile conditions after cooling to approximately 60 °C. Potato dextrose (PD) medium: Prepared using the same method as the solid medium but without the addition of agar. The culture flasks were blue-capped bottles with rubber stoppers, with a volume of 300 mL at 25 °C. Preparation of fungal inoculum plugs: Fungal mycelia were inoculated onto PDA and incubated statically in the dark at 25 °C. After the mycelia fully covered the Petri dishes, a sterile cork borer with a 1 cm diameter was used to cut out plugs containing both the medium and the attached mycelia. These plugs served as the fungal inoculum.

### 2.2. Distribution of Fixed ^13^C in Different Fungal Cellular Components

A volume of 100 mL of PD medium was added to 250 mL Erlenmeyer flasks and sterilized by autoclaving at 121 °C for 20 min. After cooling, one fungal inoculum plug (1 cm diameter) of *T. uniseriatum* was aseptically inoculated into each flask, with three replicates prepared. Under sealed conditions, the ^13^CO_2_ (99.99% Nanjing Zhongjunyuan Biotechnology Co., Ltd., Nanjing, China) concentration in each flask was adjusted to 10% (*v*/*v*). The cultures were incubated in the dark at 25 °C and 180 rpm on a shaker for 7 days. Fungal mycelia were then harvested by sterile gauze filtration for subsequent separation into cellular components (cell wall, organelles, and cytosol) and determination of ^13^C enrichment. The separation of fungal subcellular components was performed as follows: In a pre-chilled mortar at 4 °C, 0.25 g of mycelia and 8 mL of ice-cold extraction buffer were thoroughly ground into a homogenate. The homogenate was centrifuged at 3000 rpm for 15min at 4 °C. The pellet, representing the cell wall fraction, was collected. The resulting supernatant was further centrifuged at 15,000 rpm for 30 min to separate the organelle fraction (pellet) from the cytosolic fraction (supernatant). The extracts from the cell wall, organelles, and cytosol, along with the buffer control, were freeze-dried prior to ^13^C abundance analysis. The measured ^13^C value in each cellular fraction was corrected by subtracting the ^13^C enrichment value of the extraction buffer. The composition of the extraction buffer was as follows: 250 mM sucrose, 50 mM Tris-HCl (pH 7.5), and 1 mM dithioerythritol.

### 2.3. Soil Microcosm Incubation

A full-factorial design was employed, consisting of three Glucose levels (0, 100, and 200 mg·kg^−1^), three Na_2_S_2_O_3_ levels (0, 50, and 100 mg·kg^−1^), and two ^13^CO_2_ levels (5% and 10%), for a total of 18 treatments. Each treatment was replicated three times. Dry soil was aseptically weighed and evenly distributed (30 g per bottle) into the bottom of incubation bottles. The bottles were covered with breathable sealing film and sterilized twice by autoclaving at 121 °C to kill indigenous microorganisms. Subsequently, soil moisture was adjusted to 60% of the maximum water-holding capacity using sterile water. Simultaneously, one fungal inoculum plug of *T. uniseriatum* was inoculated into the center of each soil surface. The bottles were then sealed and pre-incubated for 3 days to enhance fungal acclimation to the soil environment (Figure 1). During this pre-incubation period, gas samples were collected every 12 h using a syringe, and the CO_2_ concentration was measured using a gas chromatograph (7890A, Agilent, Beijing, China). Glucose and Na_2_S_2_O_3_ were added as follows: Stock solutions of Glucose and Na_2_S_2_O_3_ were prepared separately by dissolving each in sterile water to a concentration of 30 g/L. These stock solutions were filter-sterilized through a 0.22 μm membrane filter. For each corresponding treatment, 1 mL of a mixture containing the appropriate volumes of Glucose stock solution, Na_2_S_2_O_3_ stock solution, and sterile water was applied by dripping around the periphery of the fungal colony. Following the addition, the bottles were tightly sealed. The original headspace air in the bottles was evacuated using a vacuum pump and replaced with synthetic air (CO_2_-free). This evacuation-replacement cycle was repeated twice to ensure complete displacement of the original atmosphere with CO_2_-free synthetic air. Subsequently, 15 mL or 30 mL of synthetic air was withdrawn with a syringe and immediately replaced with an equal volume of ^13^CO_2_, achieving final headspace concentrations of 5% and 10% CO_2_, respectively. The bottles were then incubated in the dark at 25 °C for 28 days under sealed conditions. Every 7 days, the headspace gas was re-equilibrated by replacing it with fresh synthetic air and readjusting the CO_2_ concentration to the target level.

At the end of the incubation period, the colony diameter of *T. uniseriatum* was measured [19], and the mycelia were collected and stored at 4 °C for subsequent determination of CO_2_ fixation enzyme activity. Prior to soil sampling, the remaining ^13^CO_2_ in the incubation bottles was evacuated using a vacuum pump. The soil samples were then processed as follows: one portion was stored at 4 °C for analysis of dissolved organic carbon (DOC), MBC, and their respective ^13^C enrichment; another portion was air-dried for determination of soil organic carbon (SOC) and ^13^C enrichment in the dry soil.

### 2.4. Enzyme Activity Assay of Phosphoenolpyruvate Carboxylase and Pyruvate Carboxylase

Mycelium (0.1 g, fresh weight) was suspended in 1 mL of ice-cold extraction buffer (50 mM 4-(2-hydroxyethyl)-1-piperazineethanesulfonic acid (HEPES), pH 7.5) and homogenized on ice. The homogenate was subjected to medium-frequency ultrasonic cell disruption (5 s on, 10 s off, 12 cycles) and centrifuged at 12,000 × *g* for 10 min at 4 °C. The resulting supernatant was collected as the crude enzyme extract and used immediately for enzymatic assays. was determined using a malate dehydrogenase (MDH)-coupled spectrophotometric assay by monitoring the oxidation rate of reduced nicotinamide adenine dinucleotide (NADH) at 340 nm. PEPC catalyzes the carboxylation of phosphoenolpyruvate (PEP) to oxaloacetate, which is subsequently reduced to malate by MDH, accompanied by the oxidation of NADH. The assay was performed following the method of Singal and Singh [21], with minor modifications. The reaction mixture (200 μL final volume) contained 50 mM Tris-HCl (pH 8.1), 5 mM PEP, 5 mM MgCl_2_, 20 mM NaHCO_3_, 0.2 mM NADH, 2 mM dithiothreitol (DTT), and approximately 0.3 U of MDH. The reaction was initiated by adding 20 μL of crude enzyme extract to 180 μL of the reaction mixture in a 96-well UV microplate. Absorbance at 340 nm was recorded at 30 °C immediately after mixing (t = 10 s) and after 5 min of reaction (t = 310 s), using a microplate reader pre-equilibrated at 30 °C. The decrease in absorbance was used to calculate the NADH oxidation rate based on the molar extinction coefficient of NADH (ε = 6.22 mM^−1^·cm^−1^). Pyruvate carboxylase (PC) activity was measured using the same MDH-coupled spectrophotometric principle as for PEPC, essentially as described by López Muñoz et al. [22]. PC catalyzes the carboxylation of pyruvate with CO_2_ to form oxaloacetate, which is subsequently reduced to malate by MDH, accompanied by the oxidation of NADH. The reaction mixture (200 μL final volume) contained 100 mM Tris-HCl (pH 8.0), 4 mM MgCl_2_, 250 mM KCl, 20 mM KHCO_3_, 4 mM ATP, 20 mM sodium pyruvate, 0.2 mM NADH, and approximately 0.4 U of MDH. The assay was performed by mixing 180 μL of the reaction mixture with 20 μL of crude enzyme extract in a 96-well UV microplate. Absorbance at 340 nm was recorded at 37 °C at t = 10 s and t = 130 s after initiation, using a microplate reader preheated to 37 °C. Enzyme activity was calculated as described for the PEPC assay. For both enzymes, one unit of activity was defined as the amount of enzyme catalyzing the oxidation of 1 nmol of NADH per minute under the respective assay conditions. Enzyme activities were normalized to mycelial fresh weight and expressed as nmol NADH consumed per gram fresh weight per minute (nmol NADH·g^−1^·min^−1^). All assays were performed in triplicate.

### 2.5. Soil Carbon Fractionation and δ^13^C Analysis

DOC and MBC were measured as follows: two 5.00 g portions of fresh soil were weighed. One portion was fumigated with chloroform, while the other served as the unfumigated control. The fumigation procedure was conducted as follows: the weighed soil sample was placed in a wide-mouthed plastic container inside a vacuum desiccator. A beaker containing anhydrous chloroform and boiling chips was placed at the bottom of the desiccator. The desiccator was sealed, and a vacuum pump was used to evacuate the air until chloroform began to boil. The vacuum was maintained for 5 min, after which the desiccator was sealed and kept in the dark at room temperature for 24 h. The unfumigated soil sample was placed in a 50 mL centrifuge tube with 20 mL of distilled water, shaken for 30 min, and then centrifuged at 4000 rpm for 5 min at 25 °C. From the resulting supernatant, 5 mL was filtered through a 0.45 μm membrane filter, and the carbon content of the extract was determined using a carbon-nitrogen analyzer (Analytik, Jena, Germany). The remaining supernatant was freeze-dried, and the resultant solid samples were analyzed for ^13^C abundance using an elemental analyzer coupled to a stable isotope ratio mass spectrometer (EA-IRMS, Thermo Scientific, Bremen, Germany). The lyophilized samples were further dried to constant weight, ground to a fine powder, and approximately 1–5 mg of each sample was weighed and encapsulated in a tin capsule. The encapsulated samples were loaded into an elemental analyzer (Flash EA 1112, Thermo Fisher Scientific, Waltham, MA, USA) for high-temperature combustion. The resulting CO_2_ was carried into a stable isotope ratio mass spectrometer (DELTA V Advantage, Thermo Fisher Scientific, Bremen, Germany) via a ConFlo IV continuous-flow interface for ^13^C abundance determination. The δ^13^C values were expressed in per mille (‰) relative to the Vienna Pee Dee Belemnite (V-PDB) standard. In each analytical batch, a reference standard (BN220389, δ^13^C = −26.27‰) was included for instrument calibration. The analytical precision, assessed using the reference standard, was better than 0.15‰, and the precision for sample analysis was better than 0.20‰. After fumigation, the fumigated soil sample was processed in the same manner, including water extraction, centrifugation, filtration, and determination of carbon content and ^13^C abundance. MBC was calculated as the difference in carbon content between the fumigated and unfumigated samples. The ^13^C abundance of MBC was calculated using the following equation:

*δ*^13^C_MBC_ = (*δ*^13^C_fumigated_ × C_fumigated_ − *δ*^13^C_unfumigated_ × C_unfumigated_)/(C_fumigated_ − C_unfumigated_)

where:

*δ*^13^C_MBC_—^13^C abundance of microbial biomass carbon;
*δ*^13^C_fumigated_/*δ*^13^C_unfumigated_—^13^C abundance of the extract from fumigated/unfumigated soil;
C_fumigated_/C_unfumigated_—carbon concentration of the extract from fumigated/unfumigated soil.

### 2.6. Statistical Analysis

One-way analysis of variance (ANOVA) followed by Tukey^’^s HSD test was performed to evaluate the differences among treatments under the same ^13^CO_2_ concentration. Multivariate ANOVA was employed to examine the main effects of individual factors and their interaction effects on the soil indexes. Data visualization was conducted using GraphPad Prism 9.5. Pearson correlation analysis was used to assess linear relationships among variables, and the resulting *p*-values were corrected using the Benjamini–Hochberg procedure to control the false discovery rate. Principal component analysis (PCA) was performed using R software v4.1 and visualized with the ggplot2 package. Random forest regression analysis was conducted using the rfPermute package [23], and the significance of each predictor’s importance was evaluated based on permutation tests (*n* = 500). Partial least squares path modeling (PLS-PM) was applied to examine the relationships between environmental factors (CO_2_, Glucose, and sulfur) [24] and fungal CO_2_ fixation based on the R package plspm [25,26].

## 3. Results

### 3.1. Fungal Adaptation to the Soil Incubation System and Subcellular Distribution of Fixed ^13^C

The adaptation time of *T. uniseriatum* was characterized by its CO_2_ release. Within 12–24 h after inoculation, the CO_2_ concentration in the sealed system showed no significant change and remained at a low level, indicating its weak metabolic activity (Figure 1A). After 24 h, the CO_2_ concentration gradually increased, exhibiting an approximately linear rise, suggesting that *T. uniseriatum* had acclimated to the environment and entered an active growth phase. The fixed ^13^C was predominantly distributed in the cell wall and organelles, while the ^13^C enrichment in the cytosol was relatively low (Figure 1B).

### 3.2. Changes in Colony Diameter and Mycelial Carbon Fixation-Related Enzyme Activities

The colony diameter of *T. uniseriatum* differed significantly across treatments (Figure 2A). In the 5% CO_2_ system, colony diameter showed a decreasing trend with increasing Glucose concentration. In the 10% CO_2_ system, the colony diameter reached its maximum when Glucose and Na_2_S_2_O_3_ concentrations were at moderate levels (100 mg·kg^−1^ Glucose, 50 mg·kg^−1^ Na_2_S_2_O_3_), indicating that this condition was most suitable for the growth of *T. uniseriatum*; however, colony diameter gradually decreased with further increases in the concentrations of both factors. Multifactorial ANOVA revealed that only Glucose had a significant effect on colony diameter (*p* = 0.01), whereas the effects of Na_2_S_2_O_3_ and CO_2_, as well as their interactions, were not significant (Figure 2A, Table 1). Regarding carbon fixation-related enzyme activities, PEPC enzyme activity increased with increasing reductive Na_2_S_2_O_3_ concentration under 10% CO_2_ conditions, with its peak appearing under the 5% CO_2_, C_100_S_50_ treatment. The Glucose, Na_2_S_2_O_3_, CO_2_, and their interactions all had highly significant effects on PEPC activity (Na_2_S_2_O_3_ × CO_2_: *p* < 0.05; all other factors and interactions: *p* < 0.001) (Table 1). PC enzyme activity was significantly higher under 5% CO_2_ conditions than under 10% CO_2_ (Student’s *t*-test, *p* < 0.001), and increased with increasing reductive sulfur concentration. The highest activity was observed under the 5% CO_2_, C_200_S_100_ treatment, while the lowest was observed under the 10% CO_2_, C_0_S_0_ treatment. The Glucose, Na_2_S_2_O_3_, CO_2_, and their interactions all had highly significant effects on PC activity (Na_2_S_2_O_3_ × CO_2_: *p* < 0.05; all other factors and interactions: *p* < 0.001) (Table 1).

### 3.3. Changes in Soil Carbon Fractions in Different Treatments

Under the 10% ^13^CO_2_ condition, the SOC content was slightly higher than that under the 5% ^13^CO_2_ (Student’s *t*-test *p* < 0.001), with the peak value observed in the 100 mg·kg^−1^ Glucose treatment and the lowest value in the treatment without Glucose addition. Under the 10% ^13^CO_2_, at a Glucose concentration of 200 mg·kg^−1^, increasing Na_2_S_2_O_3_ concentration promoted the accumulation of SOC (*p* < 0.05) (Figure 3A). The DOC content gradually decreased with increasing Glucose addition under the 5% ^13^CO_2_ (*p* < 0.05), while under the 10% ^13^CO_2_ condition, the differences in DOC content among treatments were relatively modest, statistically significant differences were still observed among certain groups (*p* < 0.05) (Figure 3B). Regarding the MBC, under the 5% ^13^CO_2_, the MBC content at a Glucose concentration of 200 mg·kg^−1^ was significantly higher than that in other treatments (*p* < 0.05). Under the 10% ^13^CO_2_ atmosphere, MBC content tended to be higher when Na_2_S_2_O_3_ was not added, but the difference did not reach statistical significance (Figure 3C). Multivariate ANOVA revealed that SOC content was significantly affected by Glucose and CO_2_ (*p* < 0.001), and all three-way and two-way interactions among the three factors were significant (*p* < 0.05) (Table 1). For DOC content, Glucose exerted a highly significant main effect (*p* < 0.001), and all interactions except Na_2_S_2_O_3_ × CO_2_ were significant (*p* < 0.05) (Table 1). For MBC content, both Glucose (*p* < 0.001) and Na_2_S_2_O_3_ (*p* = 0.003) showed significant main effects, and the interactions Glucose × Na_2_S_2_O_3_, Glucose × CO_2_, and the three-way interaction were all significant (*p* < 0.01) (Table 1).

### 3.4. Differences in δ^13^C Abundance and ^13^C Accumulation Concentration Among Different Soil Carbon Fractions

Significant differences in δ^13^C abundance were observed among different carbon fractions, and these were strongly influenced by CO_2_, Glucose, and Na_2_S_2_O_3_ (Figure 4, Table 1). Overall, the δ^13^C abundance in the active carbon fractions (DOC and MBC) was much higher than that in the relatively inert fraction SOC (Student’s *t*-test *p* < 0.001), and the ^13^C abundance in MBC was significantly higher than that in DOC (Student’s *t*-test *p* < 0.001) (Figure 4). In the 5% CO_2_ system, the addition of Glucose and Na_2_S_2_O_3_ significantly affected ^13^C abundance (*p* < 0.05), with the δ^13^C values in SOC, DOC, and MBC all being relatively high under the C_200_S_50_ treatment (Figure 4A–C). Multivariate analysis of variance (Table 1) revealed that CO_2_ had a highly significant effect on δ^13^C-SOC (*p* < 0.001), with Glucose (*p* = 0.013) and Na_2_S_2_O_3_ (*p* = 0.015) also exerting significant effects. Significant interactive effects were observed for Glucose × CO_2_ (*p* = 0.001) and Na_2_S_2_O_3_ × CO_2_ (*p* = 0.007). For δ^13^C-DOC, Glucose (*p* < 0.001), Na_2_S_2_O_3_ (*p* < 0.001), and CO_2_ (*p* = 0.040) all showed significant main effects, and all two-way interactions except Glucose × Na_2_S_2_O_3_ were significant (*p* < 0.01). Regarding δ^13^C-MBC, Glucose (*p* = 0.001) and Na_2_S_2_O_3_ (*p* = 0.008) exhibited significant main effects, while significant interactions were detected for Glucose × CO_2_ (*p* < 0.001), Glucose × Na_2_S_2_O_3_ (*p* = 0.004), and the three-way interaction (*p* < 0.001). However, the main effect of CO_2_ (*p* = 0.418) and the Na_2_S_2_O_3_ × CO_2_ interaction (*p* = 0.071) were not significant. The increase in CO_2_ concentration significantly promoted ^13^C enrichment in soil inoculated with *T. uniseriatum*. Under 10% CO_2_ conditions, the ^13^C abundance in SOC was generally higher than that under 5% CO_2_ treatments (Student’s *t*-test, *p* < 0.001). However, under the 5% CO_2_ condition, a distinct peak in ^13^C enrichment was observed in SOC and active carbon fractions under the C_200_S_50_ treatment, indicating higher CO_2_ fixation efficiency of *T. uniseriatum*. Overall, the accumulation concentration of ^13^C was highest in the SOC, while the accumulation of ^13^C in the DOC and MBC was essentially comparable (Figure 4D,E).

### 3.5. Analysis of Drivers of Fungal Carbon Sequestration Based on PCA and PLS-PM

To further elucidate the relationships among environmental factors, enzyme activities, and the accumulation of ^13^C in different soil carbon fractions, correlation analysis, random forest importance assessment, and PLS-PM analysis were conducted. PC activity was significantly and positively correlated with Na_2_S_2_O_3_ and the δ^13^C of the DOC fraction (Figure 5A, Table S1), and it also ranked high in importance in the random forest analysis (Figure 5B), suggesting that carbon fixation via the PC pathway may be more closely associated with ^13^C enrichment in DOC, and that Na_2_S_2_O_3_ might play a positive role in this process. Furthermore, PC enzyme activity was negatively correlated with CO_2_, whereas PEPC enzyme activity showed significant positive correlations with CO_2_, Na_2_S_2_O_3_, SOC concentration, the δ^13^C abundance of SOC, and the DOC. These results indicate that while Na_2_S_2_O_3_ has a generally positive promoting effect on both PC and PEPC activities, CO_2_ concentration may play a key role in regulating the dynamic balance between PC and PEPC. While Na_2_S_2_O_3_ exerts positive effects on both PC and PEPC activities, CO_2_ concentration appears to be more important in modulating the equilibrium of these two enzymes. In terms of SOC, PEPC appears to contribute more to soil carbon sequestration than PC, as reflected by its more pronounced correlations with SOC content and δ^13^C abundance, implying a closer link between the PEPC pathway and ^13^C enrichment in SOC. PCA further revealed a close association between PEPC enzyme activity and SOC content as well as its isotopic abundance (Figure 5C). Random forest modeling identified distinct sets of predictors for ^13^C accumulation in different carbon fractions (Figure 5B). For the SOC fraction, CO_2_ concentration, PEPC activity, SOC content, and its δ^13^C abundance ranked as the most important variables influencing ^13^C accumulation. δ^13^C abundance of DOC and SOC, PC, and DOC content jointly impacted ^13^C accumulation in the DOC fraction, while SOC content, MBC content and its δ^13^C abundance, PC, and Na_2_S_2_O_3_ concentration were the main factors influencing ^13^C accumulation in the MBC fraction. In the present culture system, CO_2_ was negatively correlated with PC activity and positively correlated with PEPC activity, while the addition of Na_2_S_2_O_3_ was positively correlated with both PC and PEPC activities. The PLS-PM analysis further indicated that PC activity was positively associated with ^13^C accumulation in the active carbon pools (DOC and MBC), while PEPC activity was predominantly linked to ^13^C sequestration in the relatively stable SOC pool (Figure 5D). These distinct pathway patterns suggest a functional partitioning of the two anaplerotic enzymes in directing newly fixed carbon into different soil fractions (Figure 5D).

## 4. Discussion

### 4.1. CO_2_ as a Metabolic Regulator Rather than a Growth Limiter for T. uniseriatum

In the present study, two CO_2_ concentration gradients (5% and 10%) were established based on the typical range of CO_2_ concentrations found in deep soil layers (3.5−9.2%). Within this concentration range, CO_2_ levels exhibited no significant effect on the colony diameter (growth rate) of *T. uniseriatum* (Table 1), consistent with the well-documented high tolerance of fungi to CO_2_—some fungal species can withstand concentrations as high as 36% or even 60% [18]. However, CO_2_ concentration significantly influenced the carbon fixation characteristics of *T. uniseriatum*. This suggests that, within the concentration range examined in this study, CO_2_ does not primarily function as a growth-limiting factor, but rather appears to influence carbon fixation enzyme activities, possibly through a signaling or metabolic regulatory role [20]. Previous studies have demonstrated that CO_2_ concentration profoundly affects fungal morphological differentiation and metabolic pathways. For instance, elevated CO_2_ levels can alter cell wall structure [27], while reduced CO_2_ concentrations were found to disrupt glucan metabolism and sexual differentiation in *Schizophyllum commune* [28]. More importantly, CO_2_ accumulation has been shown to induce a morphological transition from hyphal to yeast-like forms [29]. Such morphological changes could potentially modify the interface between hyphae and gaseous CO_2_, thereby affecting CO_2_ adsorption and fixation efficiency. These findings align with our previous observations that *T. uniseriatum* forms fine hyphal aggregates (<1 μm in diameter) in soil [26], a structural feature conducive to capturing high concentrations of CO_2_ within soil pores. Based on these lines of evidence, we tentatively propose that the differential fungal CO_2_ fixation characteristics observed in this study may be related to CO_2_ concentration-induced adaptive responses in hyphal morphology or microstructure, which could subsequently influence the efficiency of CO_2_ contact and fixation. Future investigations examining hyphal ultrastructure under varying CO_2_ concentrations would be helpful to test this possibility.

### 4.2. Metabolic Flexibility of T. uniseriatum: Context-Dependent Role of Thiosulfate in CO_2_ Fixation

Building upon our previous finding that sulfite promotes CO_2_ fixation in *T. uniseriatum* [15], the present study further explored the relationship between a stronger reductant (Na_2_S_2_O_3_) and the fungal CO_2_ fixation strategy. The results indicated that Glucose concentration substantially influenced the functional role of Na_2_S_2_O_3_. In the absence of Glucose, the addition of Na_2_S_2_O_3_ was associated with increased MBC content, whereas the δ^13^C value of MBC decreased. Consistent with our previous study [15], this appears to indicate that under carbon-limited conditions, the fungus preferentially utilizes Na_2_S_2_O_3_ as a sulfur source to sustain rapid growth rather than directing it toward CO_2_ fixation. Conversely, when Glucose was supplied at a sufficient concentration (200 mg·kg^−1^), a significant increase in MBC content accompanied by an elevated δ^13^C value indicated enhanced CO_2_ fixation. This indicates that under conditions where basal growth is supported by an ample carbon source, the role of Na_2_S_2_O_3_ shifts from that of a sulfur source to a donor of reducing power. Through its oxidation, Na_2_S_2_O_3_ provides the necessary energy and electrons for CO_2_ fixation processes (e.g., the reductive tricarboxylic acid (rTCA) cycle), thereby driving efficient CO_2_ fixation. This functional shift may be interpreted from a microbial physiological perspective. For oligotrophic fungi, adaptation to the culture environment or the delayed activation of CO_2_ utilization mechanisms often results in slow growth during the initial phase. Glucose, as a readily available carbon and energy source, can rapidly support basal metabolism and proliferation, which may facilitate the subsequent expression of carbon fixation-related genes. For instance, Glucose addition has been shown to significantly enhance biomass and CO_2_ assimilation rates in other autotrophic microorganisms [29]. On the other hand, Na_2_S_2_O_3_, as a favorable electron donor, exerts its effects in a manner dependent on the cellular energy status [30]. Under carbon-deficient conditions, the energy derived from its oxidation is likely directed primarily toward survival and growth; however, when carbon sources are sufficient and growth requirements are met, the excess reducing power may be increasingly channeled into CO_2_ fixation cycles. This interpretation is broadly consistent with earlier reports that thiosulfate can enhance biomass accumulation in certain fungi under carbon-limited conditions [31]. Taken together, these findings suggest that *T. uniseriatum* may employ a flexible metabolic strategy: the fungus appears to partition the metabolic flux of thiosulfate between growth and CO_2_ fixation in a manner that depends on carbon availability (e.g., Glucose concentration). Such metabolic flexibility may represent an adaptive advantage for the fungus in complex soil environments, potentially contributing to its survival and carbon sequestration capacity.

### 4.3. Differential Regulation of PEPC and PC and Their Complementary Roles in Fungal CO_2_ Fixation

In *Saccharomyces cerevisiae*, the gene encoding PEPC is repressed by Glucose [32] but induced by non-sugar compounds such as organic acids, glycerol, and amino acids [33]. In the present study, PEPC activity showed a negative correlation with Glucose concentration (Figure 4), consistent with this pattern. More notably, PEPC activity was significantly positively correlated with thiosulfate (Na_2_S_2_O_3_) concentration (Figure 4). One possible interpretation is that thiosulfate addition may promote the synthesis of sulfur-containing amino acids (methionine and cysteine) in *T. uniseriatum* [34,35] thereby indirectly upregulating PEPC activity. However, direct evidence at the transcriptional or metabolic level is needed to validate this hypothesis. Unlike PEPC, PC activity was strongly inhibited by CO_2_ but similarly stimulated by reductive sulfur (Na_2_S_2_O_3_) (Figure 4). This pattern suggests that *T. uniseriatum* may possess a thiosulfate oxidation pathway capable of extracting electrons from thiosulfate to regenerate menaquinol, NADH and reduced ferredoxin (Fdred) [36], thereby providing energy and electron support for CO_2_ fixation.

Although both PC and PEPC catalyze the formation of oxaloacetate, they differ in their energy metabolism: the conversion of phosphoenolpyruvate (PEP) to oxaloacetate by PEPC generates one ATP, whereas the PC reaction does not [16]. In the present study, PEPC activity was significantly positively correlated with ^13^C atom abundance in both SOC and DOC (Figure 4). It is conceivable that the higher energy efficiency of the PEPC reaction may contribute, at least in part, to the preferential association of PEPC activity with ^13^C enrichment in soil carbon pools, although this possibility requires further experimental validation. The two enzymes also differed in their responses to CO_2_. PEPC is generally considered an intracellular CO_2_-concentrating enzyme; previous studies have shown that in diatoms, PEPC activity is significantly higher at 0.2% CO_2_ than at ambient CO_2_ (0.042%) [37], whereas in sugarcane, PEPC activity already declines substantially at 0.072% CO_2_ [38]. In the present study, PEPC activity remained positively correlated with CO_2_ concentration even at levels as high as 10%, suggesting a relatively high CO_2_ tolerance of PEPC in *T. uniseriatum* compared with the organisms examined in the aforementioned studies. In contrast, PC activity showed a negative correlation with CO_2_ concentration, a pattern that differs from the upregulation of pyruvate carboxylase gene expression observed in microalgae under 2% CO_2_ [39], implying species- and enzyme-specific regulatory mechanisms. In summary, both anaplerotic carbon-fixing enzymes, PC and PEPC, were positively associated with reductive sulfur, but they showed contrasting responses to CO_2_: PC activity was negatively correlated with CO_2_, whereas PEPC activity remained positively correlated even at high CO_2_ levels. These contrasting patterns, together with their differing energy efficiencies, suggest a potential functional differentiation between the two enzymes. PEPC, with its higher energy yield, was more closely associated with ^13^C enrichment in soil carbon pools, while PC appeared to be more relevant under conditions of sufficient reductive sulfur and relatively low CO_2_ [40]. This apparent division of labor may represent a metabolically flexible strategy that could contribute to the adaptability of *T. uniseriatum* under varying environmental conditions, although direct evidence for this functional model remains to be established.

### 4.4. Carbon Partitioning in T. uniseriatum: Implications for Soil Carbon Stability

The fixed ^13^C was found to be predominantly distributed in the cell wall and organelles of *T. uniseriatum* (Figure 1). This distribution pattern is likely related to the relatively large contribution of cell wall material to total biomass. Similarly, A similar trend was observed in a study on *Fusarium graminearum*, in which nucleic acids showed high ^14^C fixation despite accounting for a low proportion of dry weight, while the cell wall fraction, owing to its larger biomass, became the primary site of ^14^C accumulation over time [40]. This distribution pattern has potential implications for soil carbon persistence. The cell walls of microorganisms—particularly fungi—are known to possess distinctive physical structures and chemical compositions, including β-1,4 glycosidic bonds in peptidoglycan, chitin polymers, and complexly cross-linked glycoproteins. Upon cell death, these components are generally considered to form a relatively stable and decomposition-resistant framework of microbial necromass [6]. It is therefore plausible that carbon allocated to the cell wall may have a greater potential for longer-term retention in soil, potentially contributing to the stable carbon pool. However, direct evidence for the persistence of *T. uniseriatum*-derived cell-wall carbon in soil over extended timescales was not obtained in the present 28-day experiment and awaits further investigation. Beyond the cell wall, fungal-fixed carbon may enter the soil carbon pool through additional pathways. On one hand, during senescence and apoptosis, cytoplasmic carbon can be released as DOC upon the hydrolysis of organelles; on the other hand, active secretion during growth may also serve as a source of DOC. In a previous 80-day incubation study, we observed that carbon fixed by *T. uniseriatum* was primarily retained in MBC and DOC during the early stage (10 days), but by the end of the experiment, a substantial proportion (77–82%) had been transferred to mineral-associated organic carbon (MAOC) [15]. Taken together with those earlier findings, the present 28-day results align with the short-term phase of the established long-term trajectory, where fungal-fixed carbon is actively routed into labile pools prior to potential stabilization. However, longer observations are needed to confirm whether this pattern ultimately leads to MAOC sequestration. Despite numerical variations in MBC and DOC among treatments, the overall partitioning pattern during this incubation was consistent with that short-term dynamic.

## 5. Conclusions

Taken together, the results of this study suggest that the soil fungus *T. uniseriatum* exhibits considerable metabolic flexibility in response to varying levels of CO_2_, Glucose, and thiosulfate. Within the concentration range typical of deep soil layers (5–10% CO_2_), CO_2_ appeared to act more as a metabolic signal than as a primary determinant of growth, and was differentially associated with the activities of the anaplerotic CO_2_-fixing enzymes PEPC and PC. The role of thiosulfate appeared to be context-dependent: under carbon-limited conditions, it was primarily associated with growth support (likely as a sulfur source), whereas when an adequate carbon source was available, it became more closely associated with enhanced CO_2_ fixation (potentially serving as a source of reducing power). In addition, the fixed carbon was predominantly distributed in fungal cell walls—a structural feature that may favor the formation of stable SOC pools through microbial necromass accumulation. Meanwhile, the partitioning of newly fixed carbon into active carbon pools such as DOC appeared to be associated with specific enzymatic activities, particularly that of PC. Collectively, these findings offer insights into the metabolic plasticity of free-living fungi and point to their potential relevance to soil carbon dynamics under varying CO_2_ conditions. They also suggest that fungal metabolic responses to multiple interacting environmental factors may be worth considering in future efforts to model soil carbon processes, although further studies are needed to establish the functional mechanisms and longer-term implications.

## Figures and Tables

**Figure 1:**
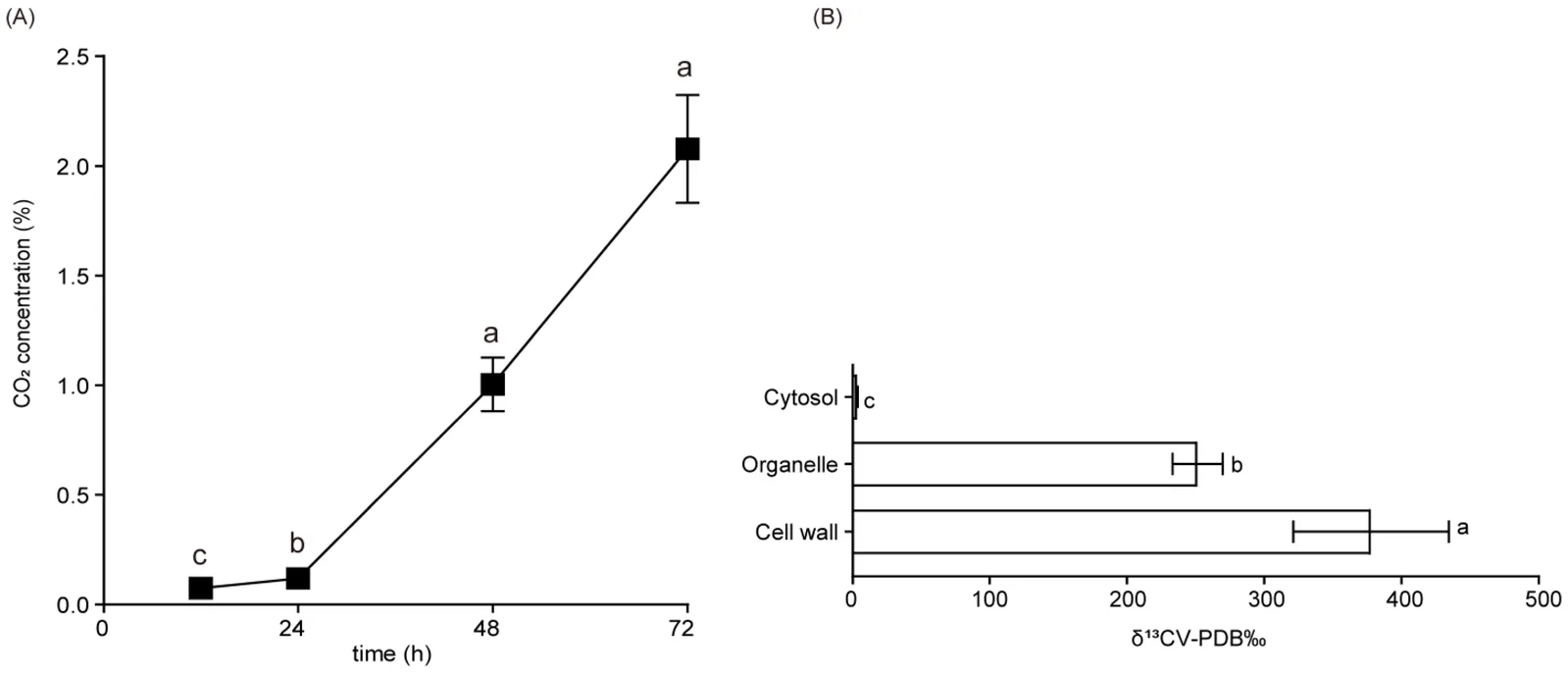
Adaptation characteristics of *Trichocladium uniseriatum* in the soil system (**A**) and subcellular distribution of its fixed ^13^C (**B**). Different lowercase letters indicate statistically significant differences among treatments (*p* < 0.05).

**Figure 2:**
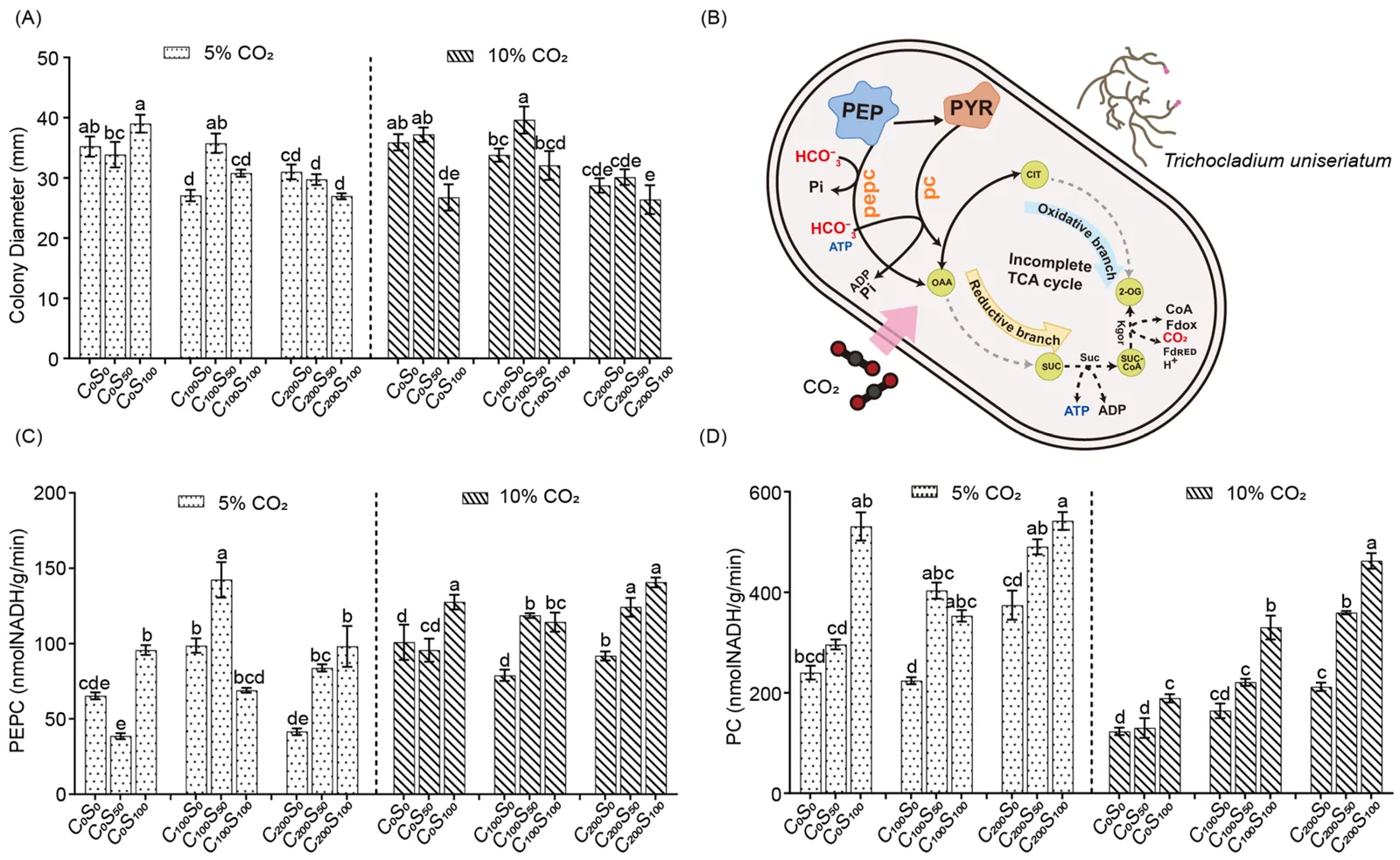
Fungal colony diameter and anaplerotic carbon fixation enzyme activities under different treatments. Different lowercase letters indicate statistically significant differences among treatments (*p* < 0.05). C and S denote Glucose and Na_2_S_2_O_3_, respectively; subscripts indicate the corresponding amendment concentrations (mg·kg^−1^ soil). For example, C_100_S_50_ represents the treatment with 100 mg·kg^−1^ Glucose and 50 mg·kg^−1^ Na_2_S_2_O_3_. (**A**) Fungal colony diameter, (**B**) schematic diagram of the catalytic reactions catalyzed by PC and PEPC, (**C**) phosphoenolpyruvate carboxylase (PEPC) activity, (**D**) pyruvate carboxylase (PC) activity.

**Figure 3:**
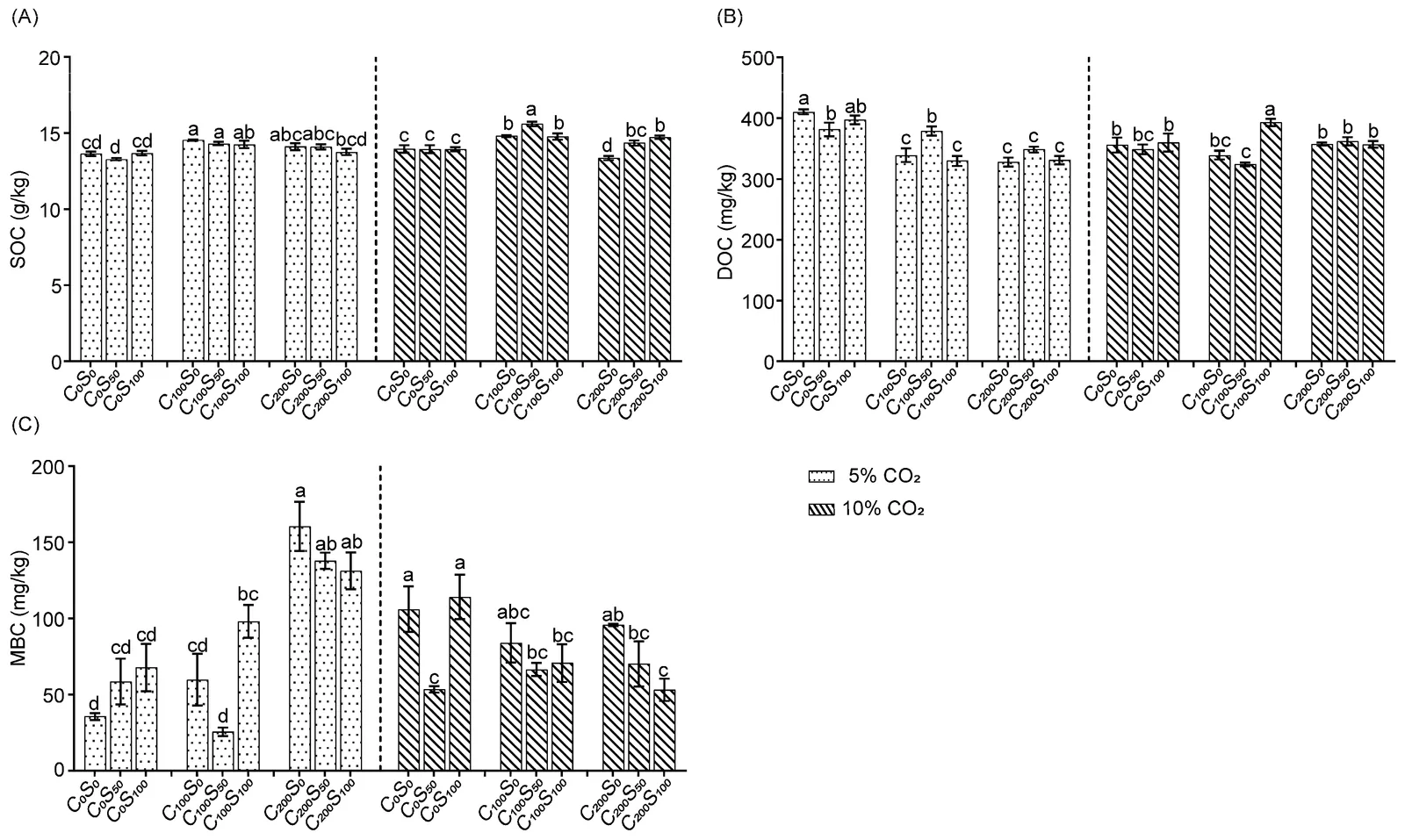
Changes in soil carbon fractions under different treatments. Different lowercase letters indicate statistically significant differences among treatments (*p* < 0.05). C and S denote Glucose and Na_2_S_2_O_3_, respectively; numerical subscripts specify the applied concentrations (mg·kg^−1^ soil). For example, C_100_S_50_ represents the treatment with 100 mg·kg^−1^ Glucose and 50 mg·kg^−1^ Na_2_S_2_O_3_. (**A**) Soil organic carbon (SOC) content, (**B**) dissolved organic carbon (DOC) content, and (**C**) microbial biomass carbon (MBC) content.

**Figure 4:**
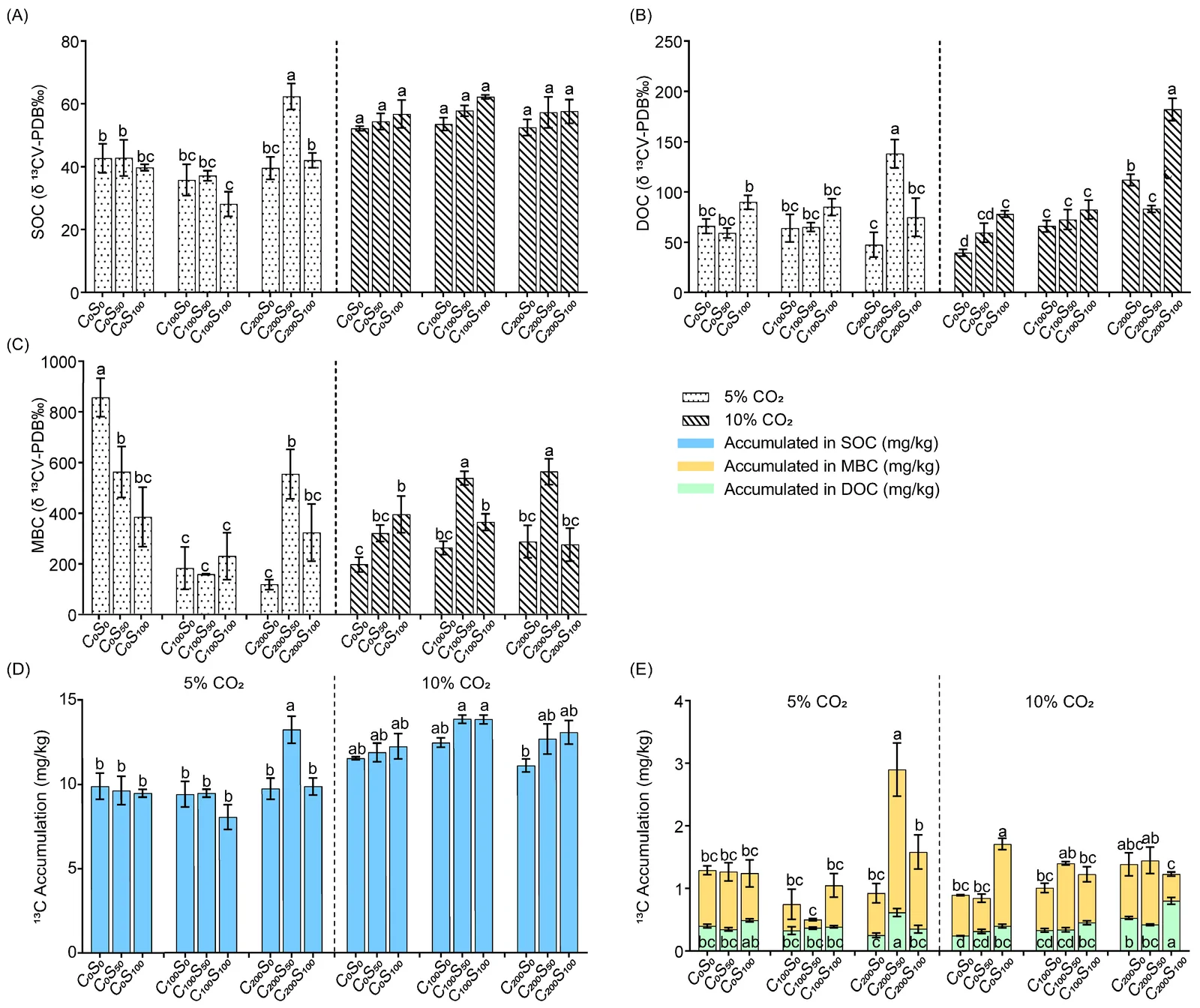
Abundance of ^13^C in soil carbon fractions and final allocation of accumulated ^13^C. Different lowercase letters indicate statistically significant differences between treatments (one-way ANOVA followed by Tukey^’^s HSD test, *p* < 0.05). C and S denote Glucose and Na_2_S_2_O_3_ additions, respectively; subscript numbers indicate the corresponding concentrations (mg·kg^−1^ soil). For example, C_100_S_50_ represents the treatment with 100 mg·kg^−1^ Glucose and 50 mg·kg^−1^ Na_2_S_2_O_3_. (**A**–**C**) δ^13^C abundance in soil organic carbon (SOC), dissolved organic carbon (DOC), and microbial biomass carbon (MBC). Final concentrations of ^13^C accumulated in SOC (**D**), DOC, and MBC (**E**).

**Figure 5:**
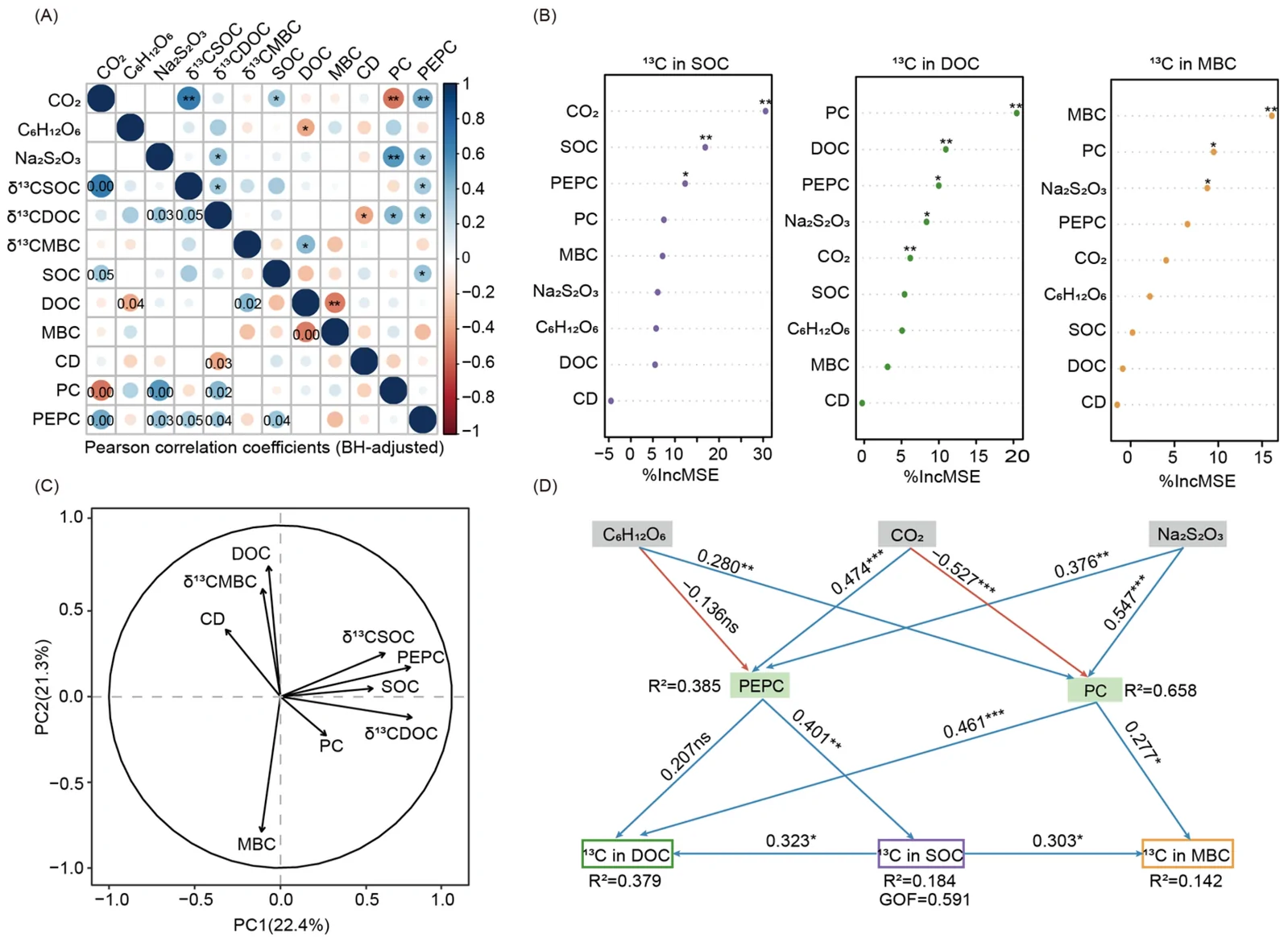
Multivariate analysis revealing the pathways of soil carbon sequestration by *Trichocladium uniseriatum*. (**A**) Pairwise Pearson’s correlations (with Benjamini–Hochberg correction). SOC, soil organic carbon; DOC, dissolved organic carbon; MBC, microbial biomass carbon; δ^13^C-SOC, ^13^C enrichment in SOC; δ^13^C-DOC, ^13^C enrichment in DOC; δ^13^C-MBC, ^13^C enrichment in MBC; CD, colony diameter; PEPC, phosphoenolpyruvate carboxylase; PC, pyruvate carboxylase. Asterisks indicate statistical significance: * *p* < 0.05, ** *p* < 0.01, *** *p* < 0.001. (**B**) Random forest analysis of factors contributing to ^13^C concentration in SOC, DOC, and MBC. (**C**) Principal component analysis of soil indexes. (**D**) Partial Least Squares Path Modeling analysis of the driving pathways of ^13^C accumulation.

**Table 1:** Effects of the three factors (Glucose, Na_2_S_2_O_3_, and CO_2_) on fungi colony diameter and soil carbon fractions.

	Factors	df	Mean Square	F Value	Significance
Colony Diameter	Glucose	2	147.192	5.205	0.01 **
	Na_2_S_2_O_3_	2	84.471	2.987	0.063
	CO_2_	1	13.835	0.489	0.489
	Glucose × Na_2_S_2_O_3_	4	34.184	1.209	0.324
	Glucose × CO_2_	2	53.798	1.902	0.164
	Na_2_S_2_O_3_ × CO_2_	2	17.480	0.618	0.545
	Glucose × Na_2_S_2_O_3_ × CO_2_	4	18.214	0.644	0.635
PEPC	Glucose	2	1212.123	10.190	<0.001 ***
	Na_2_S_2_O_3_	2	3833.108	32.224	<0.001 ***
	CO_2_	1	11,232.315	94.427	<0.001 ***
	Glucose × Na_2_S_2_O_3_	4	3449.680	29	<0.001 ***
	Glucose × CO_2_	2	2699.588	22.695	<0.001 ***
	Na_2_S_2_O_3_ × CO_2_	2	421.100	3.540	0.039 *
	Glucose × Na_2_S_2_O_3_ × CO_2_	4	1074.181	9.030	<0.001 ***
PC	Glucose	2	121,256.795	121,256.795	<0.001 ***
	Na_2_S_2_O_3_	2	143,233.093	143,233.093	<0.001 ***
	CO_2_	1	265,825.716	265,825.716	<0.001 ***
	Glucose × Na_2_S_2_O_3_	4	7460.969	7460.969	<0.001 ***
	Glucose × CO_2_	2	17,065.480	17,065.480	<0.001 ***
	Na_2_S_2_O_3_ × CO_2_	2	2664.075	2664.075	0.039 *
	Glucose × Na_2_S_2_O_3_ × CO_2_	4	15,701.014	15,701.014	<0.001 ***
SOC	Glucose	2	4.323	50.791	<0.001 ***
	Na_2_S_2_O_3_	2	0.171	2.006	0.149
	CO_2_	1	2.394	28.126	<0.001 ***
	Glucose × Na_2_S_2_O_3_	4	0.344	4.044	0.008 **
	Glucose × CO_2_	2	0.320	3.755	0.033 *
	Na_2_S_2_O_3_ × CO_2_	2	0.776	9.111	0.001 ***
	Glucose × Na_2_S_2_O_3_ × CO_2_	4	0.418	4.916	0.003 **
DOC	Glucose	2	4330.270	22.192	<0.001 ***
	Na_2_S_2_O_3_	2	184.397	0.945	0.398
	CO_2_	1	367.175	1.882	0.179
	Glucose × Na_2_S_2_O_3_	4	684.606	3.509	0.016 *
	Glucose × CO_2_	2	4849.280	24.852	<0.001 ***
	Na_2_S_2_O_3_ × CO_2_	2	1999.509	10.247	<0.001 ***
	Glucose × Na_2_S_2_O_3_ × CO_2_	4	1751.441	8.976	<0.001 ***
MBC	Glucose	2	61,103.641	22.372	<0.001 ***
	Na_2_S_2_O_3_	2	18,518.311	6.780	0.003 **
	CO_2_	1	4291.235	1.571	0.218
	Glucose × Na_2_S_2_O_3_	4	12,097.329	4.429	0.005 **
	Glucose × CO_2_	2	98,673.008	36.127	<0.001 ***
	Na_2_S_2_O_3_ × CO_2_	2	7191.423	2.633	0.086
	Glucose × Na_2_S_2_O_3_ × CO_2_	2	10,924.249	4	0.009 **
*δ*^13^C-SOC	Glucose	2	171.993	4.865	0.013 *
	Na_2_S_2_O_3_	2	166.231	4.702	0.015 *
	CO_2_	1	2999.519	84.847	<0.001 ***
	Glucose × Na_2_S_2_O_3_	4	76.320	2.159	0.093
	Glucose × CO_2_	2	317.658	8.986	0.001 ***
	Na_2_S_2_O_3_ × CO_2_	2	202.315	5.723	0.007 **
	Glucose × Na_2_S_2_O_3_ × CO_2_	4	61.694	1.745	0.162
*δ*^13^C-DOC	Glucose	2	8582.462	31.715	<0.001 ***
	Na_2_S_2_O_3_	2	4899.051	18.104	<0.001 ***
	CO_2_	1	1230.947	4.549	0.040 *
	Glucose × Na_2_S_2_O_3_	4	469.385	1.735	0.164
	Glucose × CO_2_	2	3174.111	11.729	<0.001 ***
	Na_2_S_2_O_3_ × CO_2_	2	2506.396	9.262	0.001 ***
	Glucose × Na_2_S_2_O_3_ × CO_2_	4	4192.179	15.492	<0.001 ***
*δ*^13^C-MBC	Glucose	2	125,004.084	8.694	0.001 ***
	Na_2_S_2_O_3_	2	78,825.729	5.482	0.008 **
	CO_2_	1	9664.909	0.672	0.418
	Glucose × Na_2_S_2_O_3_	4	67,422.212	4.689	0.004 **
	Glucose × CO_2_	2	282,296.890	19.633	<0.001 ***
	Na_2_S_2_O_3_ × CO_2_	2	41,010.946	2.852	0.071
	Glucose × Na_2_S_2_O_3_ × CO_2_	4	97,019.797	6.748	<0.001 ***

Asterisks denote statistical significance: * *p* < 0.05, ** *p* < 0.01, *** *p* < 0.001 (95% confidence intervals).

## Data Availability

Data will be made available on request.

## Supplementary Materials

The following supporting information can be downloaded at: https://www.mdpi.com/article/10.3390/jof12100717/s1, Table S1: Pearson correlation coefficients (*r*) and significance levels (*P*) among environmental factors, enzyme activities, and soil carbon fractions under different treatments.
