# Supplementary material for: Fungal CO2 Fixation in Soil Systems: Distinct Roles of PEPC and PC in Carbon Allocation Under Varying CO2 and Thiosulfate Conditions
Source: J Fungi (Basel). 2026 Sep 24;12(10):717. doi: 10.3390/jof12100717 (PMC13705830; doi:10.3390/jof12100717)
Supplement: Supplementary file 1 [file jof-12-00717-s001.zip › jof-4506317-supplementary.pdf]

Table S1. Pearson correlation coefficients (r) and significance levels (P) among environmental factors, enzyme activities, and soil carbon fractions under different treatments.

		CO_2	$C_6H_{12}O_6$	$Na_2S_2O_3$	^{13}CSOC	^{13}CDOC	^{13}CMBC	SOC	DOC	MBC	CD	PC	PEPC
CO_2	*P*	NA											
	r	1.000											
$C_6H_{12}O_6$	*P*	1.000	NA										
	r	0.000	1.000										
$Na_2S_2O_3$	*P*	1.000	1.000	NA									
	r	0.000	0.000	1.000									
^{13}CSOC	*P*	0.000***	0.578	0.825	NA								
	r	0.677	0.146	0.063	1.000								
^{13}CDOC	*P*	0.604	0.077	0.033*	0.050*	NA							
	r	0.135	0.313	0.378	0.341	1.000							
^{13}CMBC	*P*	0.825	0.655	0.992	0.312	0.983	NA						
	r	-0.066	-0.120	0.022	0.218	-0.026	1.000						
SOC	*P*	0.046*	0.946	0.783	0.065	0.623	0.578	NA					
	r	0.349	-0.035	0.082	0.325	0.127	-0.144	1.000					
DOC	*P*	0.759	0.037*	0.759	0.786	1.000	0.018*	0.123	NA				
	r	-0.096	-0.367	0.095	0.079	0.007	0.419	-0.284	1.000				
MBC	*P*	0.766	0.397	0.997	0.946	0.997	0.130	0.474	0.002**	NA			
	r	-0.087	0.193	-0.011	0.039	0.016	-0.276	-0.177	-0.504	1.000			
CD	*P*	0.766	0.383	0.604	0.997	0.033*	0.903	0.484	0.659	0.397	NA		
	r	0.087	-0.201	-0.133	-0.011	-0.378	0.049	0.170	0.117	-0.194	1.000		
PC	*P*	0.001***	0.125	0.001***	0.516	0.018*	0.997	0.802	0.747	0.519	0.312	NA	
	r	-0.528	0.281	0.548	-0.162	0.413	0.017	-0.074	0.102	0.159	-0.220	1.000	
PEPC	*P*	0.004**	0.604	0.033*	0.050*	0.037*	0.481	0.037*	0.946	0.113	0.762	0.825	NA
	r	0.474	-0.137	0.377	0.339	0.362	-0.173	0.361	0.036	-0.292	-0.092	0.065	1.000

Asterisks denote statistical significance: * $P < 0.05$, ** $P < 0.01$, *** $P < 0.001$ (95% confidence intervals).

		CO_2	$C_6H_{12}O_6$	$Na_2S_2O_3$	^{13}CSOC	^{13}CDOC	^{13}CMBC	SOC	DOC	MBC	CD	PC	PEPC
CO_2	*P*	NA											
	r	1.000											
$C_6H_{12}O_6$	*P*	1.000	NA										
	r	0.000	1.000										
$Na_2S_2O_3$	*P*	1.000	1.000	NA									
	r	0.000	0.000	1.000									
^{13}CSOC	*P*	0.000***	0.578	0.825	NA								
	r	0.677	0.146	0.063	1.000								
^{13}CDOC	*P*	0.604	0.077	0.033*	0.050*	NA							
	r	0.135	0.313	0.378	0.341	1.000							
^{13}CMBC	*P*	0.825	0.655	0.992	0.312	0.983	NA						
	r	-0.066	-0.120	0.022	0.218	-0.026	1.000						
SOC	*P*	0.046*	0.946	0.783	0.065	0.623	0.578	NA					
	r	0.349	-0.035	0.082	0.325	0.127	-0.144	1.000					
DOC	*P*	0.759	0.037*	0.759	0.786	1.000	0.018*	0.123	NA				
	r	-0.096	-0.367	0.095	0.079	0.007	0.419	-0.284	1.000				
MBC	*P*	0.766	0.397	0.997	0.946	0.997	0.130	0.474	0.002	NA			
	r	-0.087	0.193	-0.011	0.039	0.016	-0.276	-0.177	-0.504	1.000			
CD	*P*	0.766	0.383	0.604	0.997	0.033*	0.903	0.484	0.659	0.397	NA		
	r	0.087	-0.201	-0.133	-0.011	-0.378	0.049	0.170	0.117	-0.194	1.000		
PC	*P*	0.001***	0.125	0.001***	0.516	0.018*	0.997	0.802	0.747	0.519	0.312	NA	
	r	-0.528	0.281	0.548	-0.162	0.413	0.017	-0.074	0.102	0.159	-0.220	1.000	
PEPC	*P*	0.004**	0.604	0.033*	0.050*	0.037*	0.481	0.037*	0.946	0.113	0.762	0.825	NA
	r	0.474	-0.137	0.377	0.339	0.362	-0.173	0.361	0.036	-0.292	-0.092	0.065	1.000
